# Preservation of Non-nidal Medial Veins During Microsurgical Resection of Cerebral Arteriovenous Malformations: Technical Considerations and Early Clinical Outcomes

**DOI:** 10.7759/cureus.116079

**Published:** 2026-09-10

**Authors:** Md. Moshiur Rahman, Robert Ahmed Khan, Md. Rokibul Islam, Muhtamim Chowdhury, Md. Mahbub Hasan

**Affiliations:** 1 Department of Neurosurgery, Holy Family Red Crescent Medical College Hospital, Dhaka, BGD; 2 Department of Neurosurgery, Bangladesh Medical University, Dhaka, BGD; 3 Department of Neurosurgery, Bangladesh Medical College, Dhaka, BGD; 4 Department of Neurosurgery, Sir Salimullah Medical College and Mitford Hospital, Dhaka, BGD

**Keywords:** cerebral avm, digital subtraction angiography, medial veins, microsurgical resection, modified rankin scale, promis, seizure outcomes, spetzler-martin grade, venous infarction, venous preservation

## Abstract

Background

The surgical management of cerebral arteriovenous malformations (AVMs) requires a meticulous balance between complete nidus eradication and the preservation of adjacent normal physiological structures. While the elimination of true malformed, nidal-draining veins is mandatory to avoid intraoperative rupture, sacrificing adjacent, non-malformed, medial cortical, and deep venous channels can cause localized venous congestion, tissue infarction, and poor functional outcomes.

Objective

This study analyzed the clinical, radiological, and functional outcomes of a deliberate microsurgical strategy focused on preserving critical medial venous pathways during AVM resection in an early patient series.

Materials and methods

A retrospective study was conducted on a consecutive series of 16 adult patients (aged >18 years) who underwent micro-neurosurgical resection of cerebral AVMs (Spetzler-Martin grades I-IV) at a single private academic institution between 2018 and 2025. Preoperative baseline features, clinical presentation (hemorrhage, seizures, focal deficits), and structural profiles were documented. Microsurgical dissection emphasized circumferential nidal isolation with strict preservation of non-nidal medial veins. Long-term functional assessment was evaluated using the Patient-Reported Outcomes Measurement Information System (PROMIS) score and the modified Rankin Scale (mRS) at a minimum of six months postoperatively.

Results

A total of 16 patients were included in the study, of whom 10 were male (62.5%) and six were female (37.5%). Primary clinical presentations included intracranial hemorrhage in 10 patients (62.5%), seizures in eight patients (50.0%), and focal deficits in nine patients (56.3%). Complete angiographic eradication of the AVM nidus was achieved in all 16 patients. No perioperative mortality was observed. Postoperative outcome assessment demonstrated improvements or stabilization in functional capacity. Favorable neurological recovery, defined as mRS 0-2, was documented in 14 patients (87.5%), supported by increases in PROMIS physical and mental health T-scores. No delayed venous infarction or hemorrhagic transformation occurred in any patient.

Conclusions

In this early clinical series, prioritizing the integrity of non-malformed medial veins during cerebral AVM surgery was a safe, effective, and anatomically sound strategy. Preserving healthy venous pathways surrounding the malformation limits the incidence of secondary venous hypertension, leading to exceptional functional preservation and minimal morbidity across a range of Spetzler-Martin grades.

## Introduction

Cerebral arteriovenous malformations (AVMs) are complex congenital or dynamic vascular anomalies characterized by a high-flow, low-resistance shunt connecting feeding arteries directly to draining veins without an intervening capillary bed [1-4]. The primary management objective for symptomatic AVMs is the complete obliteration of the vascular nidus to eliminate the long-term risk of catastrophic intracranial hemorrhage, which carries substantial morbidity and mortality [5]. Among the validated treatment options, micro-neurosurgical resection remains the definitive modality for immediate and durable curative elimination of low- to intermediate-grade lesions [6,7].

Classic neurosurgical paradigms emphasize the early isolation of feeding arterial pedicles, circumferential dissection around the nidal margins within the gliotic plane, and late ligation of the primary draining veins. However, the complex vascular anatomy of AVMs, particularly those located in medial, deep parietal, or occipital regions, often features a shared or highly compact venous architecture where normal parenchymal veins lie adjacent to or are displaced by the high-flow malformed channels [8]. During aggressive circumferential skeletonization of the nidus, these non-nidal medial venous pathways may be susceptible to mechanical manipulation, unintended bipolar coagulation, or surgical sacrifice [9,10].

Sacrificing healthy venous pathways that drain normal eloquent brain tissue can lead to localized venous hypertension, tissue edema, delayed venous infarction, or secondary hemorrhagic conversion [11]. Furthermore, clinical outcome documentation in historical neurosurgical series has heavily relied on clinician-centered metrics such as the modified Rankin Scale (mRS) [12]. While the mRS provides an assessment of gross disability, it lacks the granularity required to evaluate multidimensional, patient-centered variables. Contemporary clinical research therefore increasingly incorporates validated patient-reported outcome measures, such as the Patient-Reported Outcomes Measurement Information System (PROMIS), to provide a broader representation of postoperative recovery [13].

This study presents an outcome analysis of an early consecutive series of patients undergoing microsurgical AVM resection with deliberate preservation of non-nidal medial veins. The primary objective was to describe the technical feasibility, perioperative safety profile, and early functional outcomes associated with this microsurgical strategy, without implying comparative efficacy relative to alternative venous management approaches.

## Materials and methods

This was a retrospective, single-center clinical cohort study at Comfort Nursing Home, Dhaka, Bangladesh, that included consecutive adult patients who underwent microsurgical resection for cerebral AVMs between January 2018 and July 2025, with follow-up data collected through December 2025. The study was reviewed and approved by the Institutional Review Board of Comfort Nursing Home (reference number: Comfort/IRB/2026/3F; dated July 10, 2026).

Eligibility criteria

Patients were required to have adequate documented clinical and radiological postoperative follow-up, with approximately six months or longer of follow-up available for inclusion in the outcome analysis. Inclusion criteria were age greater than 18 years at presentation, radiologically confirmed cerebral AVM, direct open microsurgical resection, and availability of complete clinical and postoperative radiological outcome data. Patients were excluded if they were aged 18 years or younger, were treated exclusively with stereotactic radiosurgery or endovascular embolization without subsequent microsurgical resection, had incomplete medical records, or lacked adequate postoperative follow-up. The inclusion and exclusion criteria are summarized in Table 1. A total of 16 patients fulfilled the criteria and were included in the study.

**Table 1:** Inclusion and exclusion criteria AVM: arteriovenous malformation

Inclusion Criteria	Exclusion Criteria
Age >18 years	Age ≤18 years
Radiologically confirmed cerebral AVM	Incomplete clinical or radiological records
Underwent microsurgical AVM resection	Treated only with embolization or radiosurgery without subsequent microsurgical resection
Available preoperative and postoperative clinical data	Lost to adequate follow-up
Available postoperative radiological confirmation	No postoperative outcome data

Preoperative assessment and grading

All patients underwent preoperative neuroimaging assessment, including structural brain magnetic resonance imaging (MRI) and vascular imaging, to characterize the nidus, associated parenchymal changes, arterial supply, and venous drainage pattern. Formal cerebral digital subtraction angiography (DSA) was used when available to delineate feeding arteries, nidal architecture, and superficial and deep venous pathways.

AVMs were classified according to the Spetzler-Martin grading system based on nidus size, eloquence of adjacent brain tissue, and venous drainage pattern. Baseline clinical characteristics, including presenting hemorrhage, seizure history, and focal neurological deficits, were obtained from the available medical records.

Surgical technique and medial vein management

All microsurgical procedures were performed by the same senior neurosurgeon using microsurgical magnification and standard micro-instruments. The operative exposure was individualized according to lesion location, vascular anatomy, and the relationship of the nidus to adjacent eloquent structures. For example, the case illustrated in Figure 1 was approached through a right parietal craniotomy.

**Figure 1:**
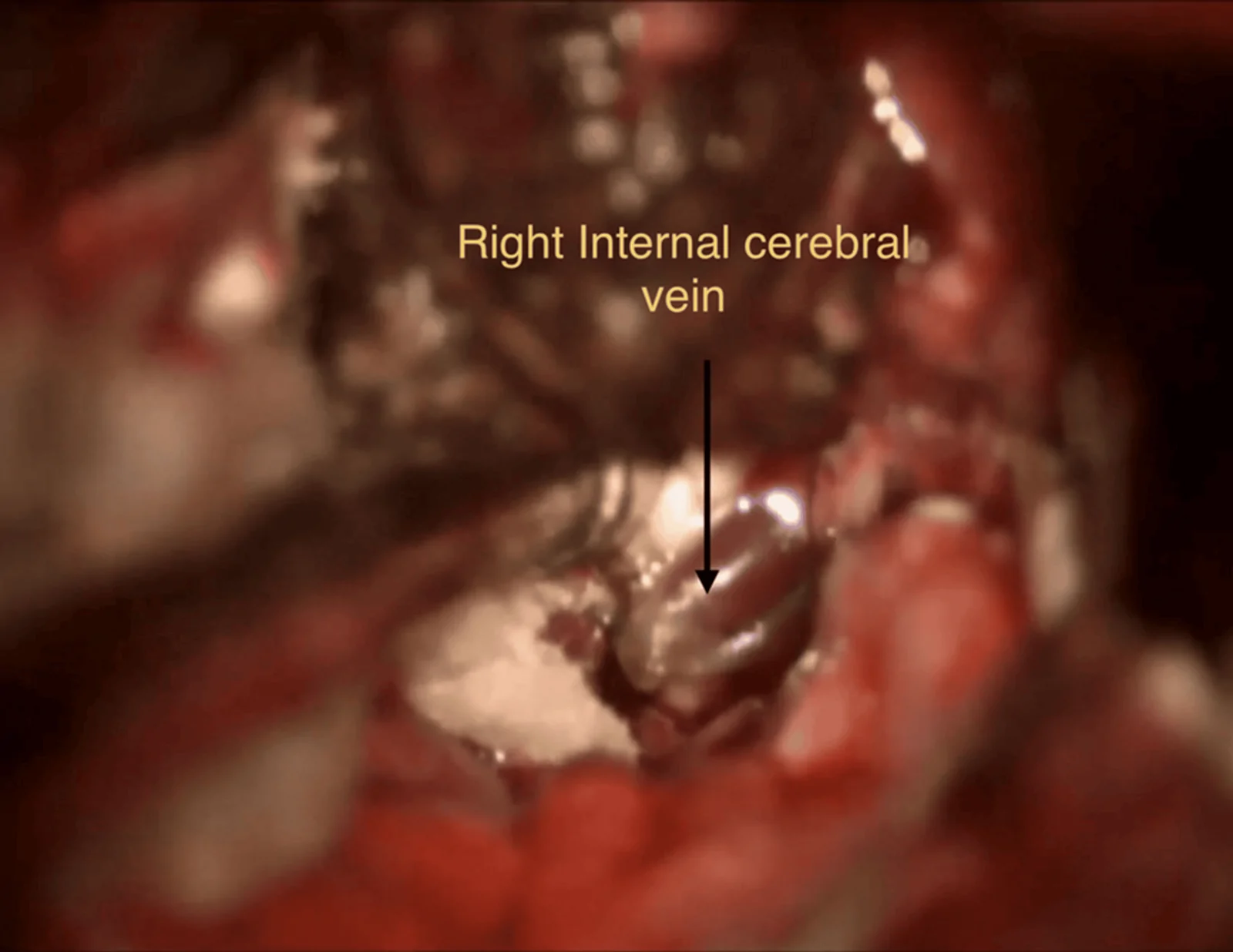
Intraoperative view obtained through a right parietal craniotomy during microsurgical resection of a centro-parietal arteriovenous malformation, showing preservation of the venous structure (arrow) identified as the right internal cerebral vein along the medial operative field.

For the purposes of this study, “non-nidal medial veins” were defined as medial or deep venous structures draining normal brain parenchyma rather than functioning as the primary venous outflow from the AVM nidus. These structures included the internal cerebral vein, basal vein of Rosenthal, medial cortical tributary veins, and other relevant deep venous pathways encountered during dissection.

In cases associated with parenchymal hemorrhage, the hematoma cavity was entered when clinically appropriate to decompress adjacent tissue and facilitate exposure of the nidus. Microsurgical dissection was performed along the gliotic plane surrounding the malformation. Arterial feeders were identified and sequentially disconnected close to the nidal margin using microsurgical clips or bipolar coagulation.

During circumferential dissection, particular attention was directed toward distinguishing high-flow nidal draining veins from venous channels draining normal brain tissue. This distinction was based on preoperative angiographic anatomy, anatomical trajectory, relationship to the nidus, and direct microsurgical inspection. Venous structures considered to drain normal brain tissue were preserved whenever technically feasible and protected from unnecessary manipulation or coagulation.

Bipolar coagulation of a venous structure was avoided until its relationship to the nidus and drainage pattern had been sufficiently assessed. Following arterial disconnection and circumferential isolation of the nidus, the primary nidal draining vein was divided, and the malformation was removed.

Preservation of non-nidal venous pathways was assessed intraoperatively by direct microscopic visualization and subsequently evaluated using postoperative vascular imaging. Because of the retrospective design, additional intraoperative flow-assessment techniques could not be uniformly reconstructed across all cases.

Outcome measures and data analysis

The primary radiological outcome was complete AVM obliteration on postoperative vascular imaging. Postoperative vascular assessment was performed using CT angiography (CTA) or DSA according to the available clinical record. CTA and DSA were treated as distinct vascular imaging modalities in the interpretation of postoperative radiological outcomes. Safety outcomes included perioperative mortality, new permanent neurological deficits, postoperative venous infarction, hemorrhagic complications, and other clinically documented postoperative adverse events.

Functional status was assessed using the mRS and was categorized as favorable functional status (mRS 0-2) or unfavorable functional status (mRS 3-5). Patient-reported physical and mental health outcomes were assessed using the PROMIS Global Health Scale v1.2 (PROMIS-10) [14]. Physical health and mental health summary scores were expressed as standardized T-scores.

Because this was a retrospective study, the original timing and mode of administration of baseline PROMIS assessments were not uniformly documented in the available records. Baseline PROMIS findings were therefore interpreted cautiously because of the potential for recall and documentation bias, and changes in PROMIS T-scores were not interpreted as clinically meaningful solely on the basis of numerical increase. Where appropriate, it was considered in relation to published minimally important difference thresholds for the relevant PROMIS domains.

Due to the limited sample size of this early series (n=16), statistical analysis was descriptive. Continuous variables were summarized using means with ranges or standard deviations, as appropriate, while categorical variables were reported as frequencies and percentages. No multivariable analysis was performed because of the small cohort size.

## Results

Demographic and clinical characteristics

A total of 16 patients were included in the study. Of these, 10 were male (62.5%), and six were female (37.5%). The mean age of the cohort was 34.5 ± 11.2 years (range: 19-58 years). Regarding clinical presentation, 10 patients (62.5%) were recorded as having intracranial hemorrhage, eight patients (50.0%) had a seizure disorder, and nine (56.3%) had focal neurological deficits. The Spetzler-Martin grade distribution included Grade I in three patients (18.8%), Grade II in five patients (31.3%), Grade III in six patients (37.5%), and Grade IV in two patients (12.5%). Deep venous drainage was present in eight patients (50.0%). Baseline demographic and clinical characteristics are summarized in Table 2.

**Table 2:** Baseline demographic and clinical characteristics of the patient cohort (N=16)

Parameter	Subcategory	Frequency	Percentage
Sex	Male	10	62.5
	Female	6	37.5
Age	Mean ± SD	34.5 ± 11.2 years	—
	Range	19-58 years	
Clinical presentation	Intracranial hemorrhage	10	62.5
	Seizure disorder	8	50.0
	Focal neurological deficit	9	56.3
Spetzler-Martin grade	Grade I	3	18.8
	Grade II	5	31.3
	Grade III	6	37.5
	Grade IV	2	12.5
Venous drainage	Deep venous drainage present	8	50.0

Surgical and radiological outcomes

Complete AVM obliteration was documented in all 16 patients (100%) on postoperative vascular imaging using CTA or DSA. Non-nidal medial and deep venous structures identified during operative planning and dissection were preserved at the conclusion of surgery. Figure 1 demonstrates the intraoperative appearance of the venous structure identified as the right internal cerebral vein.

There were no cases of intraoperative mortality or late perioperative death. No patient experienced delayed postoperative intraparenchymal hemorrhage or hemorrhagic transformation associated with acute venous outflow restriction. Furthermore, postoperative imaging showed no evidence of focal venous infarctions or progressive perilesional edema in the territories drained by the preserved medial veins. Representative postoperative vascular imaging findings are shown in Figures 2, 3.

**Figure 2:**
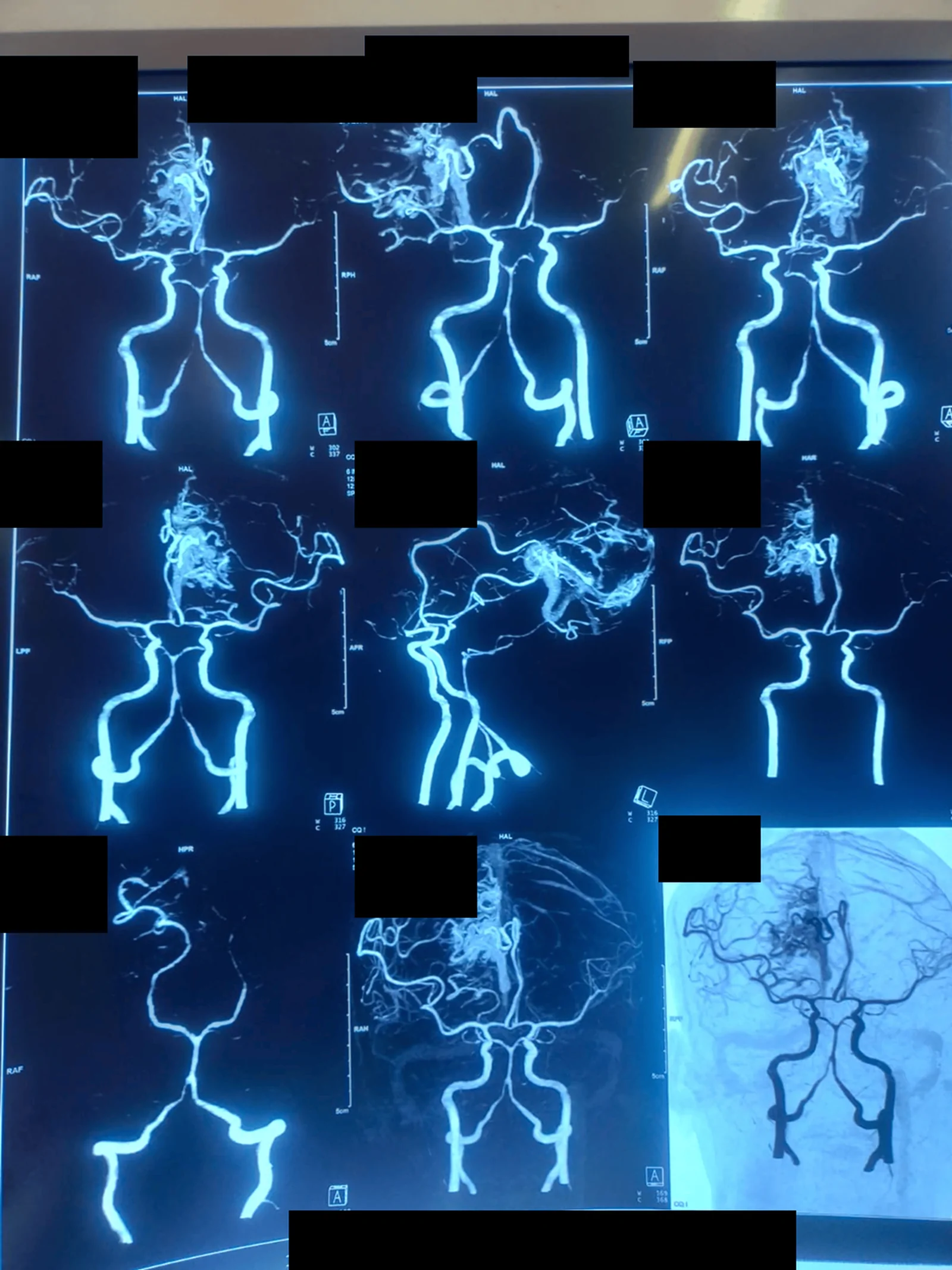
CT angiography images showing the vascular architecture of a right centro-parietal AVM involving anterior and posterior circulation vessels with associated deep venous drainage. AVM: arteriovenous malformation

**Figure 3:**
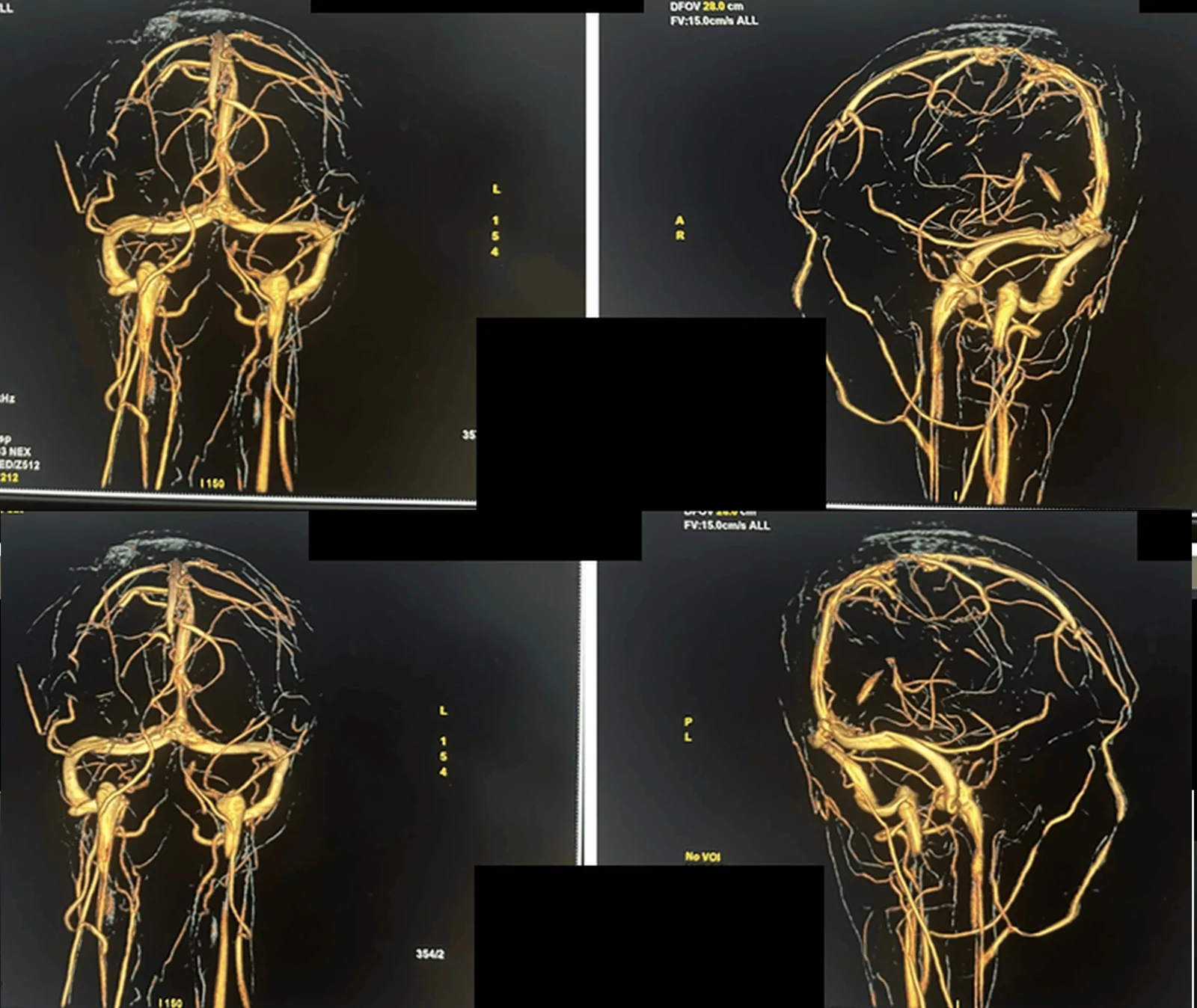
Three-dimensional CT angiography reconstruction showing a right centro-parietal AVM with complex superficial and deep venous drainage. AVM: arteriovenous malformation

Complications

No perioperative deaths were documented in the cohort. No delayed postoperative intraparenchymal hemorrhage or hemorrhagic transformation was observed. Postoperative imaging demonstrated no focal venous infarction or progressive perilesional edema in the territories associated with the preserved medial venous pathways. No new permanent neurological deficit attributable to postoperative venous infarction was documented during the available follow-up period.

Functional and patient-reported outcomes

The mean follow-up duration was 14.2 ± 6.8 months (range: 6-28 months). At the final follow-up, 14 patients (87.5%) had a favorable functional status, defined as an mRS score of 0-2. Among the 10 patients presenting with ruptured AVMs, eight (80.0%) changed from dependent baseline status to independent functional status during follow-up. Preoperative and postoperative mRS scores are shown in Figure 4.

**Figure 4:**
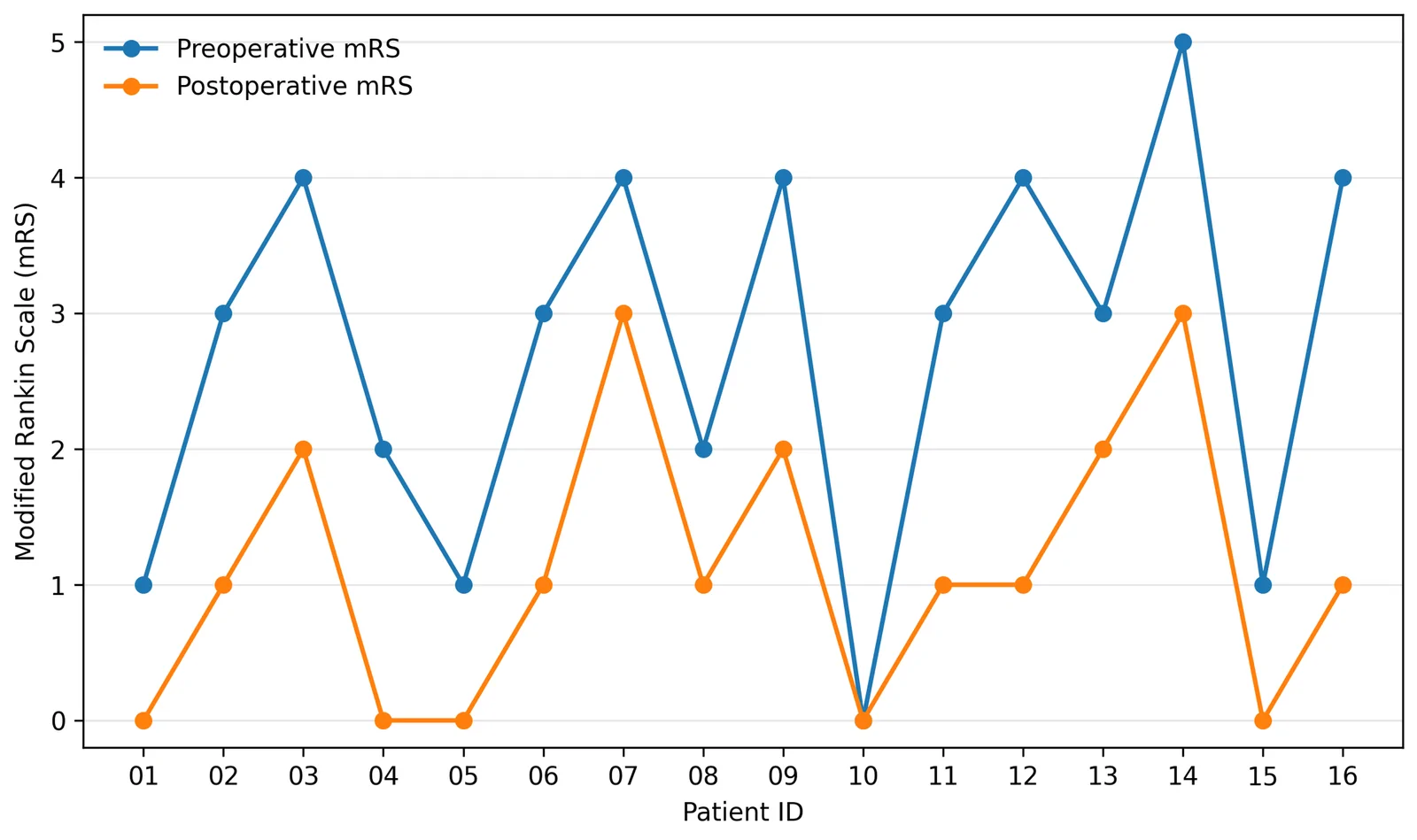
Preoperative and postoperative modified Rankin Scale (mRS) scores in the study cohort.

Of the nine patients with pre-existing focal neurological deficits, six (66.7%) had complete resolution, two (22.2%) had mild residual deficits, and one (11.1%) remained stable. Among the eight patients presenting with active seizures, six (75.0%) were documented as seizure-free during follow-up, while two (25.0%) had a greater than 75% reduction in reported seizure frequency.

Available PROMIS Physical Health T-scores increased from a mean pre-surgical value of 38.4 ± 5.2 to 52.1 ± 4.8 at follow-up, corresponding to a mean numerical increase of 13.7 T-score points. PROMIS Mental Health T-scores increased from 41.2 ± 6.1 to 54.6 ± 5.3, corresponding to a mean numerical increase of 13.4 T-score points. These findings are presented descriptively and should be interpreted cautiously given the retrospective nature of baseline PROMIS assessment and the potential for recall and documentation bias. Numerical changes were not considered clinically meaningful solely on the basis of an increase in T-score and should be interpreted in relation to established minimally important difference thresholds for the relevant PROMIS domains. Changes in PROMIS Physical Health and Mental Health T-scores are shown in Figure 5. Individual patient-level functional and patient-reported outcome data are presented in Table 3.

**Figure 5:**
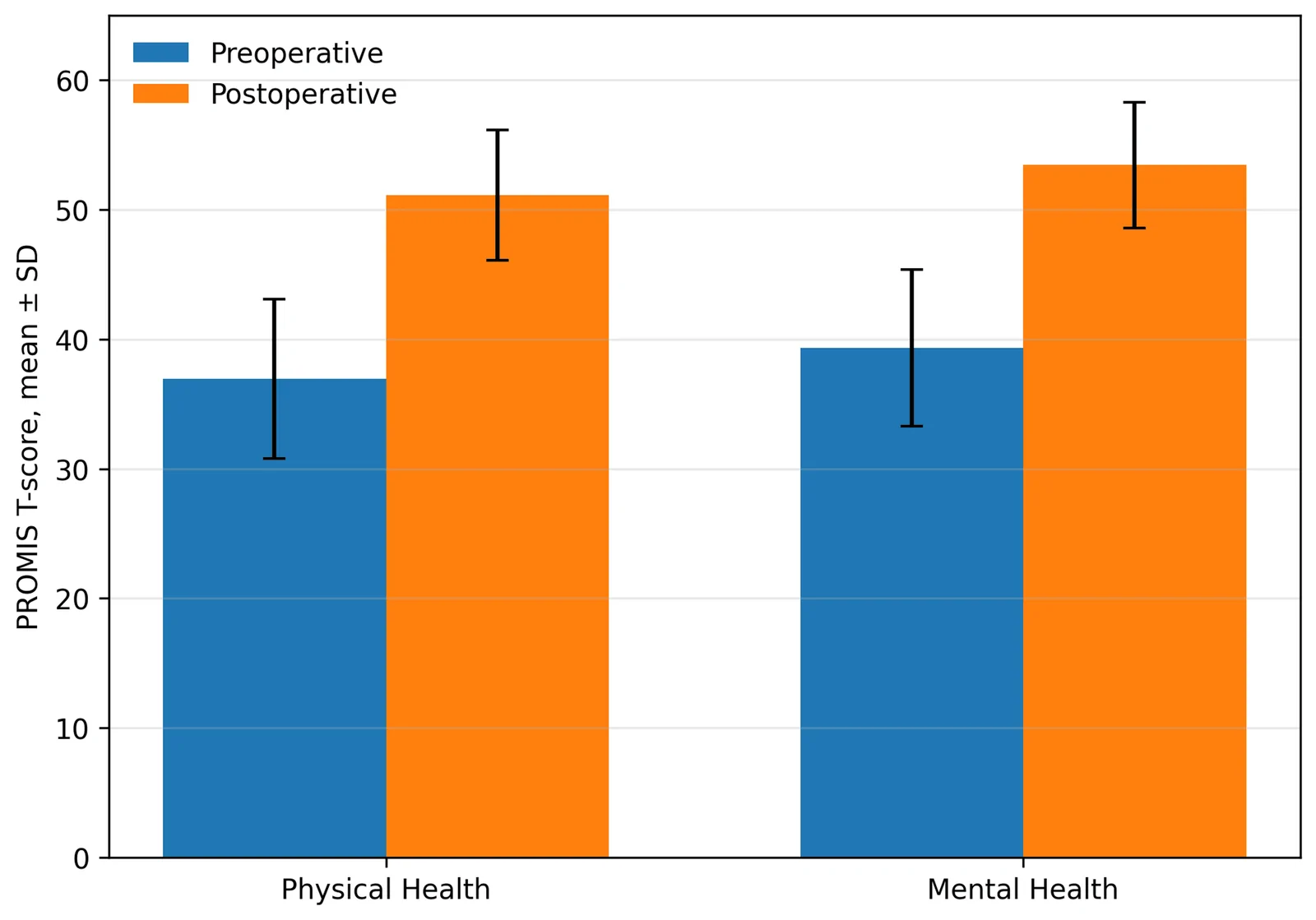
Preoperative and postoperative PROMIS Physical Health and Mental Health T-scores in the study cohort. PROMIS: Patient-Reported Outcomes Measurement Information System

**Table 3:** Individual patient functional and patient-reported outcome trajectories (N=16) mRS: modified Rankin Scale; PROMIS: Patient-Reported Outcomes Measurement Information System; SM: Spetzler-Martin

Patient ID	SM Grade	Preoperative mRS	Postoperative mRS	Preoperative PROMIS (Physical/Mental)	Postoperative PROMIS (Physical/Mental)	Clinical Resolution Status
01	I	1	0	44 / 46	55 / 58	Complete seizure resolution
02	II	3	1	35 / 38	52 / 54	Hemorrhage recovery; deficit resolved
03	III	4	2	31 / 35	48 / 51	Hemianopia partially improved
04	II	2	0	42 / 45	56 / 55	Seizure free
05	I	1	0	45 / 48	58 / 60	No new deficits
06	III	3	1	36 / 40	50 / 53	Motor deficit fully resolved
07	IV	4	3	30 / 32	42 / 44	Stable motor deficit; independent walk
08	II	2	1	40 / 41	54 / 56	Seizure frequency minimized
09	III	4	2	33 / 36	49 / 52	Hematoma cleared; independent
10	I	0	0	48 / 50	56 / 57	Incidental finding; intact
11	III	3	1	37 / 39	51 / 55	Resolution of speech hesitancy
12	II	4	1	32 / 34	53 / 56	Favorable hematoma recovery
13	III	3	2	35 / 37	47 / 50	Walk independently with minor limp
14	IV	5	3	26 / 28	40 / 42	Improved from bedridden to assisted
15	II	1	0	43 / 44	55 / 58	Seizure free
16	III	4	1	34 / 36	52 / 54	Full sensory deficit recovery

## Discussion

The principal goal of microsurgical resection of cerebral AVMs is complete exclusion of the vascular nidus while preserving surrounding functional brain tissue and normal vascular structures. Although established microsurgical principles emphasize sequential arterial feeder control and delayed division of the primary draining vein, the management of adjacent non-nidal venous pathways may be particularly challenging in lesions with compact or deep venous anatomy [10,15]. In this early series of 16 patients, deliberate preservation of non-nidal medial venous pathways was technically feasible and was associated with complete AVM obliteration, no perioperative mortality, and no documented postoperative venous infarction. However, because this was an uncontrolled retrospective series, these outcomes should be interpreted as descriptive associations rather than evidence that venous preservation independently produced the observed clinical results.

The potential importance of preserving normal venous drainage is supported by the hemodynamic characteristics of cerebral AVMs. Abnormal arteriovenous shunting alters local blood flow and venous pressure relationships, while normal parenchymal veins may lie adjacent to, or be displaced by, malformed high-flow channels [16,17]. Consequently, inadvertent injury to venous pathways draining normal brain tissue could theoretically impair local venous outflow and contribute to venous congestion, edema, infarction, or secondary hemorrhagic complications [18]. These technical considerations may be especially relevant in ruptured AVMs, where hematoma, tissue distortion, and reactive gliosis can make identification of normal venous anatomy more difficult [19].

The operative strategy used in this series emphasized preservation of venous structures considered to drain normal brain parenchyma. Microsurgical dissection was maintained close to the gliotic nidal boundary, arterial feeders were sequentially disconnected, and coagulation of venous channels was avoided until their relationship to the nidus had been adequately assessed. In cases associated with intraparenchymal hemorrhage, hematoma evacuation was used when clinically appropriate to improve operative exposure. No postoperative venous infarction or clinically documented delayed venous congestion was observed in this cohort. Nevertheless, in the absence of a comparator group in which comparable non-nidal venous structures were sacrificed, the present study cannot determine whether preservation itself was responsible for the absence of these complications.

The study also incorporated patient-reported outcome measures alongside the mRS. At final follow-up, 14 of 16 patients (87.5%) had an mRS score of 0-2. Numerical increases were also observed in PROMIS Physical Health and Mental Health T-scores, which increased from 38.4 ± 5.2 to 52.1 ± 4.8 and from 41.2 ± 6.1 to 54.6 ± 5.3, respectively. These changes should not be interpreted as proof of a treatment effect, particularly because baseline PROMIS assessment was reconstructed retrospectively and is therefore susceptible to recall and documentation bias. Interpretation of PROMIS changes should also consider established minimally important difference thresholds for the relevant instrument and domains rather than numerical change alone.

Among the eight patients presenting with active seizures, six were documented as seizure-free during follow-up, and two had a greater than 75% reduction in reported seizure frequency. These findings are clinically relevant descriptive outcomes; however, the study design does not permit attribution of postoperative seizure control specifically to preservation of medial venous pathways. Seizure outcomes may have been influenced by several factors, including complete nidal resection, resolution of hemorrhage-related irritation, lesion location, antiseizure treatment, and other perioperative variables.

Overall, the present findings support the technical feasibility of identifying and preserving non-nidal medial venous pathways during microsurgical AVM resection. They should be regarded as hypothesis-generating rather than confirmatory. Larger prospective studies with standardized imaging, patient-reported outcome assessment, longer follow-up, and appropriate comparator groups are required to determine whether deliberate preservation of these venous structures independently influences neurological and patient-reported outcomes.

Limitations

This study has several important limitations. First, it represents a small, single-center retrospective cohort of only 16 patients. The limited sample size reduces statistical power, limits generalizability, and precludes meaningful multivariable analysis of factors independently associated with outcome. The absence of a comparator group is particularly important because the study cannot establish a causal relationship between preservation of non-nidal medial veins and the favorable neurological or radiological outcomes observed.

Second, selection bias is possible because operative strategy and technical decisions were individualized according to lesion anatomy and intraoperative findings. Lesions in which venous preservation was technically achievable may differ systematically from lesions in which such preservation would be difficult or impossible. Although the single-surgeon design provides technical consistency, it may also limit generalizability to other surgeons and institutional settings.

Third, patient-reported outcomes were assessed using the PROMIS-10. However, because of the retrospective study design, the original timing and mode of administration of baseline PROMIS assessments were not uniformly documented. Baseline PROMIS findings are therefore susceptible to recall and documentation bias, particularly among patients presenting with acute hemorrhage or neurological impairment.

Fourth, postoperative outcomes were not assessed through a formally blinded or independently adjudicated process in all cases, introducing the possibility of observer bias. CTA and DSA were also used as distinct postoperative vascular imaging modalities according to the available clinical records, and their diagnostic characteristics are not identical.

Fifth, detailed patient-level lobar location and laterality were not consistently available in the retrospective dataset used for this analysis. Consequently, anatomical subgroup analyses according to AVM lobe or hemisphere could not be performed.

Sixth, patient enrolment extended through July 2025 and follow-up data were collected through December 2025. Follow-up duration therefore varied among patients, and longer surveillance is required to characterize the durability of seizure control, functional recovery, and delayed vascular or neurological complications.

Finally, this early series does not establish deliberate preservation of non-nidal medial venous pathways as a standard of care. Larger prospective, multicenter studies incorporating standardized vascular imaging, standardized PROMIS administration, independent outcome assessment, longer follow-up, and an appropriate comparison group are required to determine the reproducibility and independent clinical value of this microsurgical strategy.

## Conclusions

This early clinical series suggests that preservation of non-nidal medial venous pathways during microsurgical resection of cerebral AVMs is technically feasible and can be incorporated into a structured microsurgical workflow. In this cohort of 16 patients, complete nidus obliteration was achieved without perioperative mortality or documented postoperative venous infarction, and favorable functional and patient-reported outcomes were observed during follow-up. However, the small sample size, retrospective single-center design, absence of a comparator group, and limitations in retrospective outcome assessment preclude conclusions regarding the independent effect of venous preservation on clinical outcomes. These findings should therefore be considered hypothesis-generating. Larger prospective, multicenter studies with standardized vascular imaging, patient-reported outcome assessment, independent outcome adjudication, and appropriate comparison groups are needed to determine the reproducibility and clinical value of this microsurgical strategy.
